# The More, the Merrier…? Antipsychotic Polypharmacy Treatment Strategies in Schizophrenia From a Pharmacology Perspective

**DOI:** 10.3389/fpsyt.2021.760181

**Published:** 2021-11-24

**Authors:** Stephan Hjorth

**Affiliations:** ^1^Department of Molecular and Clinical Medicine, Institute of Medicine, The Sahlgrenska Academy at Gothenburg University, Gothenburg, Sweden; ^2^Pharmacilitator AB (Inc.), Vallda, Sweden

**Keywords:** antipsychotics, polypharmacy, schizophrenia, pharmacodynamic profiles, efficacy, adverse events, drug combinations, pros and cons

## Abstract

Antipsychotic polypharmacy/drug combination treatment (APP) is a remarkably common practice in the schizophrenia context, given the lack of general support in treatment Guidelines. There is also a vast literature on APP outcomes, but a paucity of high-quality evidence-based data to guide and optimize adequate use of APP. This seems particularly true regarding many pharmacology-based considerations involved in APP treatment strategies. This paper first briefly summarizes clinical literature related to the use of APP. Against this backdrop, the pharmacological target profile features are then described of frequently used antipsychotic agents, in relation to estimated free plasma exposure levels at clinically efficacious dosing. APP strategies based on the properties of these drugs are then scrutinized and gauged within the background literature framework. The anticipated usefulness of APP from the pharmacological standpoint is detailed regarding efficacy, adverse effect (AE)/tolerability, and safety perspective, including why, when, and how it may be used to its advantage. For the purpose, a number of theoretically beneficial combinations as well as instances with suboptimal—and even futile—APP approaches are exemplified and discussed from the rational pharmacodynamic and pharmacokinetic pros and cons point-of-view. In this exposé, particular attention is paid to the utility and features of 3rd Generation Antipsychotic dopamine (DA) D2-D3 agonists within an APP setting.

## Introduction

In an ideal pharmacotherapy setting, schizophrenia treatment with a single antipsychotic agent would be preferable. The treatment drug should additionally be broadly efficacious across symptom domains, while devoid of patient tolerability, safety, and adverse effect issues—thereby overall promoting medication adherence and quality of life. Needless to say, this is however far from the real-world experience with pharmacological treatment approaches to schizophrenia.

**Figure 1:**
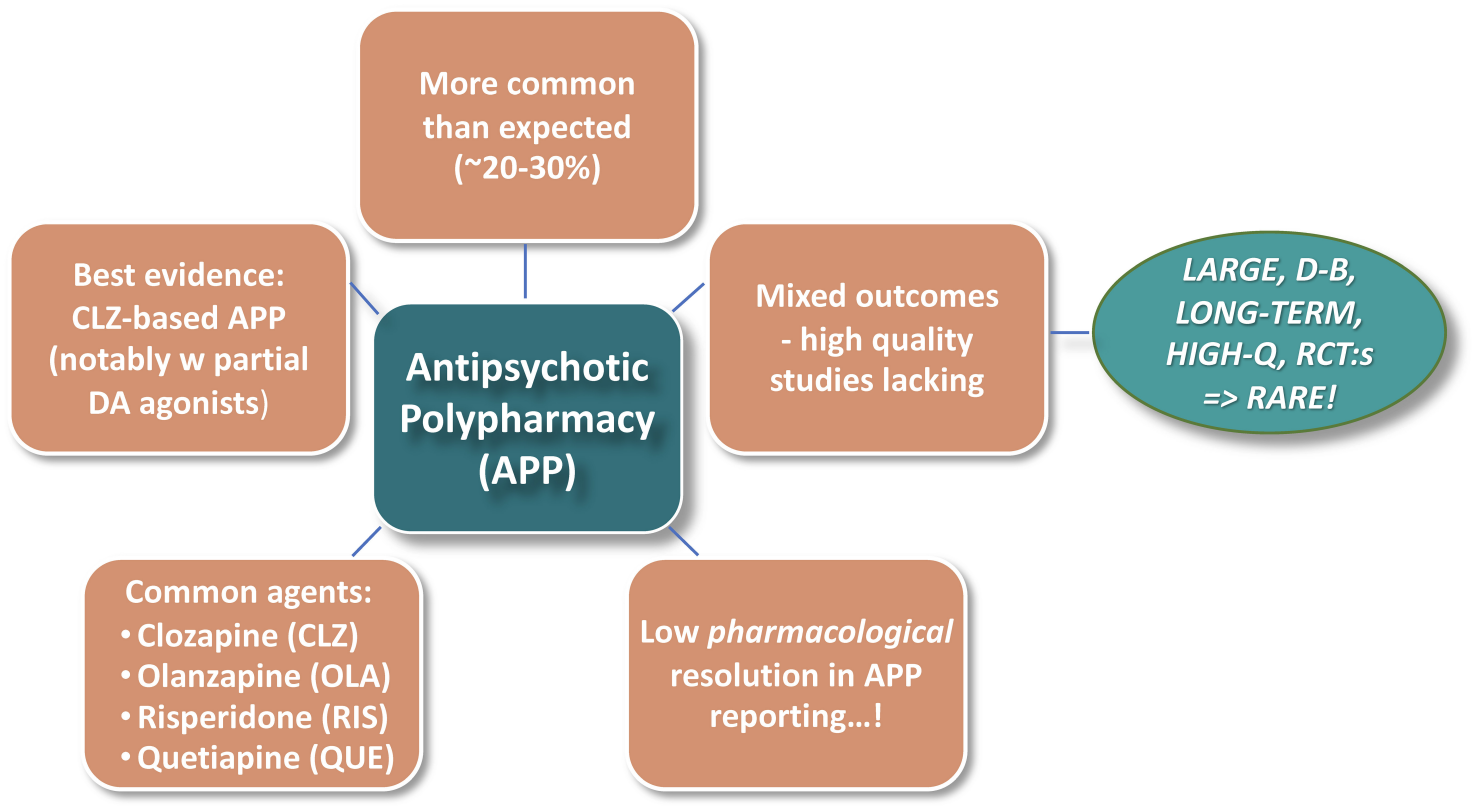
Antipsychotic polypharmacy (APP)—broad literature background impressions.

Figure 1 illustrates some general background impressions from the—vast—Antipsychotic PolyPharmacy (APP) literature. Notwithstanding that the practice is not generally encouraged by treatment Guidelines, APP is remarkably common in the schizophrenia context. Reported rates also vary substantially across geographies, timeframes, and treatment conditions, with recent median prevalence figures, at least in Western societies, ranging typically between 20 and 30% (1–3). However, there is a paucity of evidence-based data to guide and optimize adequate use of APP. This seems particularly true regarding many pharmacology-based considerations involved in APP strategies.

A comprehensive formal review of the practice of APP *per se* is beyond the scope of the current paper, where the prime focus is upon pharmacological underpinnings in relation to the diverging outcome of combinations of different antipsychotic agents. With a view to nonetheless set a relevant framework for the discussions, the current account attempts to assess and synthesize background knowledge from the APP literature, with particular attention paid to pharmacological aspects involved. This framework is to a large extent based on meta-analysis studies and authoritative systematic reviews (1–4) but also includes information extracted from searches on single agents in the polypharmacy-in-schizophrenia context.

## APP: Why, When, and to Whom?

**Figure 2:**
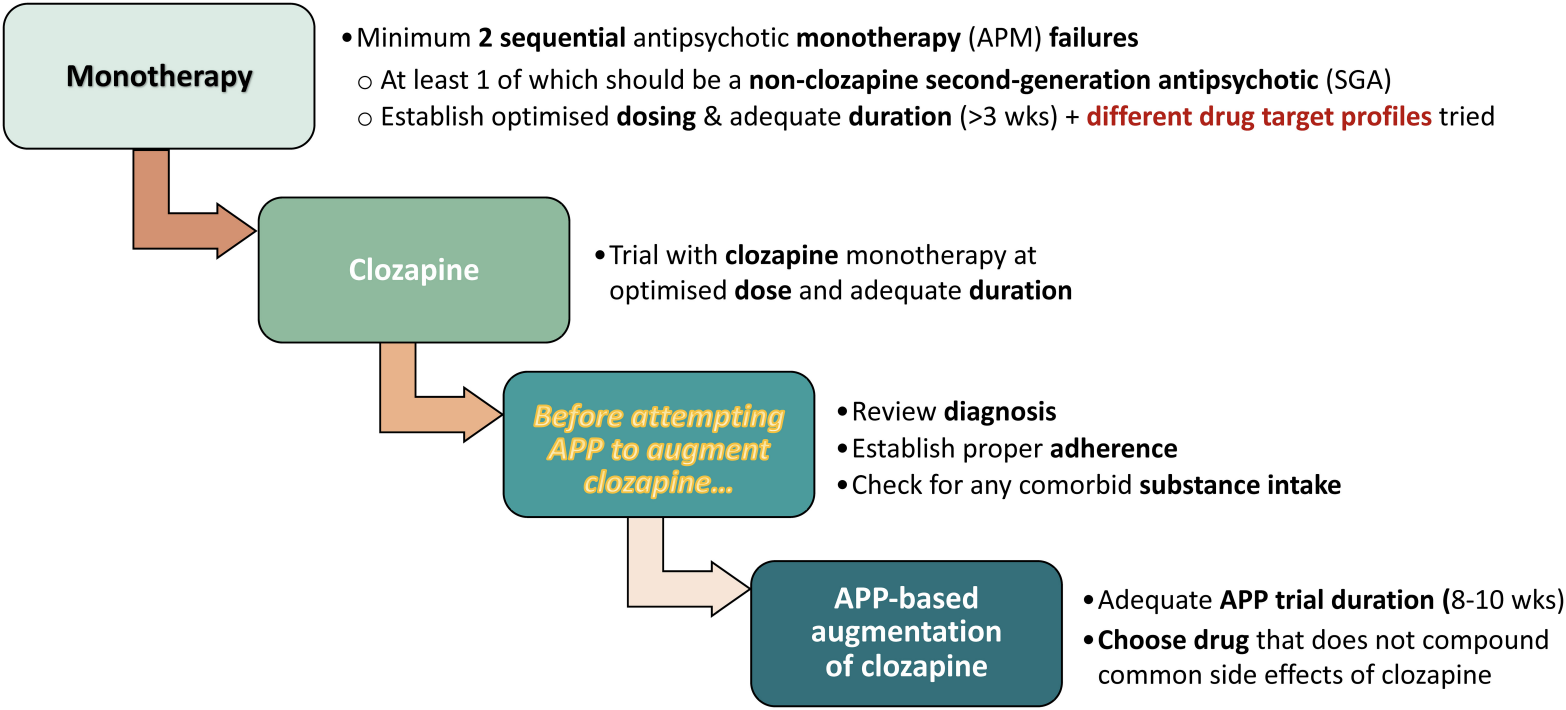
Stepwise transition to APP, based on NICE, UK, Clinical Guidelines (5).

Clearly, APP is not for every single antipsychotic therapy situation. The directions described in the NICE, UK, Clinical Guidelines (5) may be viewed as a prototypical example on when to—potentially—introduce APP. Briefly, this represents a stepwise transition from 2 or more failed antipsychotic monotherapy (APM) trials, through clozapine (CLZ; monotherapy) treatment, and then onwards to third-line APP therapy approaches (with proper control assessment stations on the way), and the explicit recommendation to take pharmacological differences in antipsychotic drug profiles into account (see, Figure 2).

A variety of reasons for instigating APP have been given. These include general desires to enhance, broaden, and sustain treatment efficacy—but also to attenuate adverse events (AE; e.g., weight gain, metabolic issues, and prolactin rise) and as a preventive measure vs. relapse and rehospitalization—relative to the outcome from APM alone. The aforementioned ambitions apply in particular concerning the management of negative and cognitive symptoms, in patients with greater illness severity and complexity, longer duration of illness and hospitalization, and treatment refractoriness (1, 6, 7). Other associations of APP, e.g., with younger age and male sex may stem from more severe (negative) symptoms already at early age in males than in females (7). Observed variations across geographies possibly reflect an impact of local therapy tradition and inherited prescriptions (1).

### Commonly Given Pharmacodynamic (PD) Reasons

**Figure 3:**
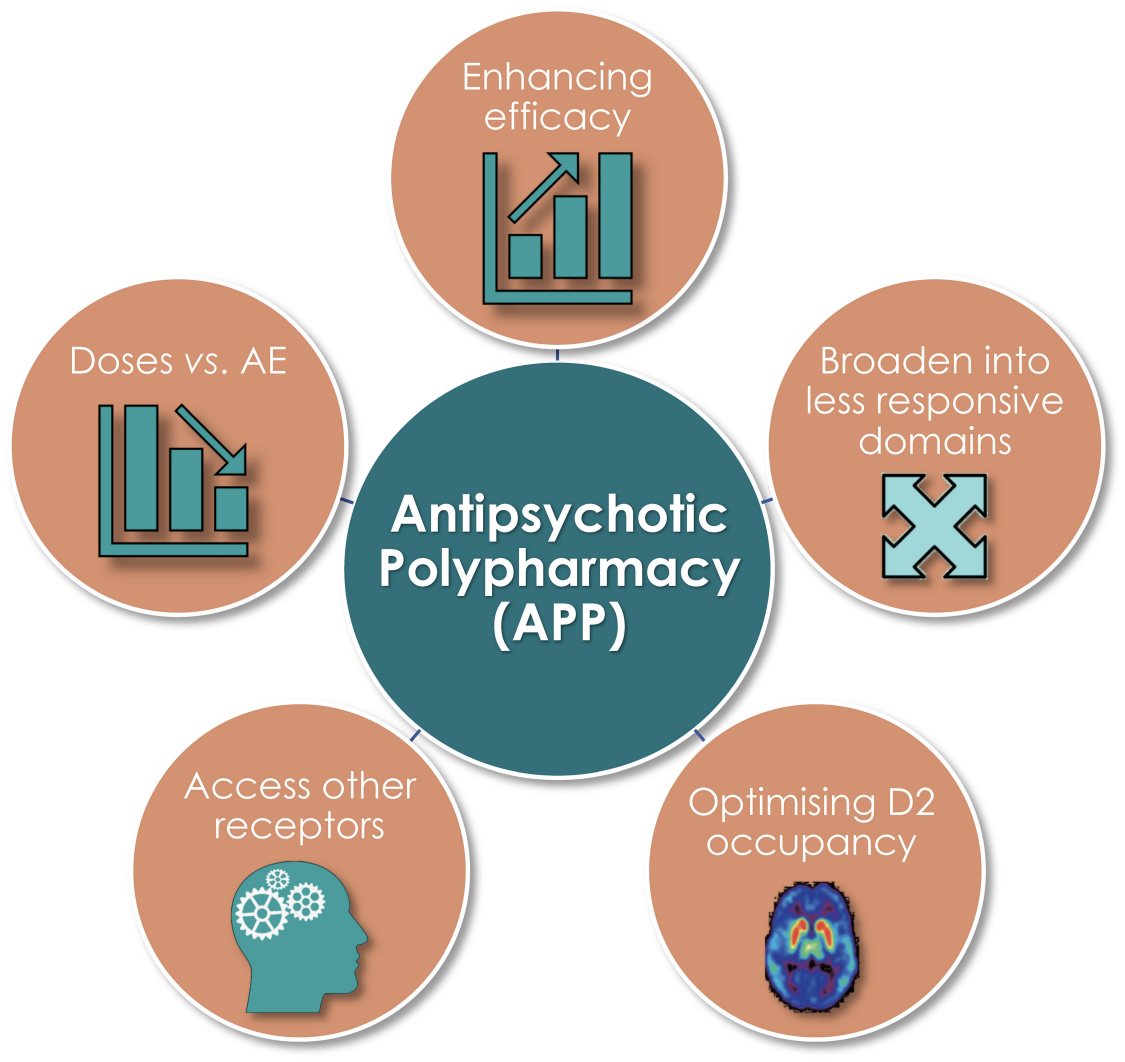
Commonly given pharmacodynamically (PD)-based reasons to initiate APP.

Several pharmacodynamically-based—and inter-related—reasons for initiation of APP have been cited in the literature [Figure 3; (1, 8)]. Among these, unsurprisingly, an aim to enhance efficacy and broaden clinical effect into less responsive symptom domains is commonplace, as is the intent to adjust antipsychotic dose vs. adverse event issues (AE). The more direct target-focused reasons include a desire to optimize D2 receptor occupancy, and/or to achieve an overall more favorable treatment response (efficacy and/or AE) outcome by pharmacologically accessing other receptor categories and/or subtypes.

### Concerns, Questions, and Considerations

A number of concerns with APP have also been raised (1, 9). Some of the more common actually contrast with the stated intentions for APP. For example, published data suggest that APP often implies higher (instead of lower) total dosages of antipsychotics and increased (rather than attenuated) risks for AE (1, 10, 11)—tentatively related to increased *total* antipsychotic dosage. With more than one drug on board, there might also be a greater risk of drug-drug interaction events, and difficulties attributing a beneficial or undesired response to the individual antipsychotic agent in an APP treatment combination.

Against the above backdrop it appears reasonable to ask whether APP is

- *Effective…?*
- *Tolerable…?*
- *Safe…?*
- *Useful for relapse, re-hospitalization, and prevention purposes?*
- *Based on clear pharmacological rationale…?*

### APP: Commonly Used Agents?

Which antipsychotics are common in APP contexts then? In the literature [e.g., (12, 13)], recurrently emerging drug choices for APP are first- and second-generation (FGA and SGA, respectively) antipsychotic agents like haloperidol (HAL), olanzapine (OLA), risperidone (RIS), quetiapine (QUE), and clozapine (CLZ). In addition, the earliest of the third generation antipsychotics (TGA), aripiprazole (ARI), appears to be a common APP add-on choice to many of the above FGA and SGA class drugs [see, e.g., (14)]. A number of recent case reports likewise suggest that cariprazine (CAR) may emerge as a beneficial option in the APP context [(15–18); *vide infra*], whereas so far there does not seem to be any reports of the use of the latest TGA brexpiprazole in APP combination approaches. A brief overview of antipsychotic target profiles toward APP is shown in Table 1 below.

**Table 1:** Examples of antipsychotics considered in the APP context.

**Drug**	**Desired target/s and affinity**	**Adverse effect (AE) target/s**	**Key property**
Haloperidol (HAL)	Strong D2	D2	Antagonist
Olanzapine (OLA)	5-HT2A/modest D2	H1, 5-HT2C, cholinergic, D2	Antagonist
Risperidone (RIS)	5-HT2A/D2	H1, D2, alpha1	Antagonist
Quetiapine (QUE)	5-HT2A/poor D2	alpha1, H1, cholinergic	Antagonist
Clozapine (CLZ)	5-HT2A/poor D2	H1, 5-HT2C, alpha1, cholinergic	Antagonist
Aripiprazole (ARI)	Strong D2/D3	D2?	Partial agonist
Cariprazine (CAR)	Strong D3/D2	D2?	Partial agonist

### Efficacy of APP vs. Monotherapy?

A recent review and meta-analysis of the literature (4) found superiority of APP vs. monotherapy in open-label, low-quality studies. However, method-related factors and confounders limited the generalizability of interpretations and conclusions, and in corresponding high-quality, double-blind, randomized studies there was support for enhanced efficacy only for selected APP strategies *vs*. monotherapy. Specifically, while no superiority was found for combinations of two FGA/SGA D2 antagonists, addition of the partial DA agonist antipsychotic ARI to CLZ medication significantly improved negative symptoms compared to CLZ alone. Given the basic pharmacology profile of CAR it appears reasonable to assume that this agent would work at least equally well to ARI as adjunct to CLZ. Actually, recent case reports concurs with this suggestion [(16, 17); *vide infra*], although larger, high-quality, double-blind, randomized studies will be needed for further substantiation.

This said, in schizophrenia the identification of factors at the *individual patient level* will be particularly important to increase the chance of success with an APP approach, thereby promoting personalized treatment in this very heterogeneous patient population. *In short:* finding the “right drug combination” to the “right patient” is key!

### Tolerability and Safety of APP?

While tolerability and safety concerns may cause some hesitancy to start APP, it should be noted that “*…not all antipsychotic combinations are created equal”* (8). In addition, it is not the APP approach *per se* that is the issue, but rather *the composition of the specific agents and doses comprised therein* that matter. In this regard, a systematic review and meta-analysis of randomized controlled trials comparing APP and monotherapy found no differences regarding intolerability-related treatment discontinuation (4). Moreover, again attesting to the above, it is more likely that APP strategies involving a greater total antipsychotic dose, and thus net target (e.g., D2 receptor) occupancy (8)—at least by antagonist agents [see, e.g., (19)]—would be more liable to increase the AE burden. Conversely, therapeutically useful effects on AE outcome may be achieved by partial DA agonist add-on to CLZ, RIS, OLA, or HAL, to relieve metabolic- and prolactin (PRL)-derived issues (*vide infra*). Although so far available data are insufficient to allow definitive conclusions it would appear that—contrary to what may be widely presumed—there is *a priori no general* tolerability, AE, or safety [including mortality; e.g., (20, 21)] *reason* to discard APP as a possible strategy for a patient in need thereof.

### Which Targets Are Key for Antipsychotic Drug Benefit and AE?

The action of agents in the antipsychotic class must be gauged against their individual pharmacologic target profiles, as many carry multiple receptor affinities and activities.

The dopamine (DA) D2 receptor is a pharmacological target shared by all antipsychotics in current use. However, the affinity for the target varies considerably among drugs in the class—from very high in, e.g., HAL, to pretty poor in, e.g., CLZ and QUE. Moreover, mechanistically the TGA agents ARI and CAR act as *partial agonists* rather than full antagonists at the D2, D3, and 5 HT1A receptor sites.

In addition to the above, the majority of FGA and SGA also act as blockers of several other neuroreceptor sites with clinical bearing. Table 2 lists key antipsychotic targets and clinically observed outcomes (desired and AE) associated with antagonism or partial agonist drug action at the corresponding sites.

**Table 2:** Key antipsychotic targets + associated drug benefit and AE impact examples.

**Target**	**Clinical effects associated with antagonism or partial agonism**	
	**Desired**	**Adverse effects (AE)**
D2(*)	Antipsychotic *(positive symptoms)*	EPS, prolactin ↑, sexual dysfunction and cognition ↓
D3*	Antipsychotic *(negative symptoms)*	
5-HT1A*	Anxiolytic, antidepressant, anti-EPS(?)	
5-HT2A	Anti-EPS and –akathisia	
5-HT2C		Appetite/weight ↑ and metabolic effects
H1	Sedation	Sedation, cognition ↓, appetite/ weight ↑
Alpha1		Hypotension, sexual dysfunction
Muscarinic	Anti-EPS	Dry mouth, constipation, blurry vision, cognition ↓

* *Partial agonism*

(*) *Antagonism (Desired and Adverse Effects) or partial agonism (only Desired)*.

## Pharmacological Profiles of Antipsychotics in APP Endeavors

In an aim to provide an easily and rapidly accessible overview of overall target profile patterns of the various antipsychotics discussed “cobweb” diagrams were generated by means of the polar chart diagram function in Microsoft Excel. The “cobweb” approach was employed also as a means to graphically illustrate the array of differences and similarities among antipsychotics commonly used in APP combinations and to further enhance the relevance by integrating into the graphs, depictions of corresponding clinically efficacious unbound drug exposures (further details, see Figure legends). These diagrams thus show the pharmacological fingerprint (drug affinities) vs. approximate free plasma exposures at steady-state and clinically efficacious dosing of the antipsychotics discussed in further detail below.

**Figure 4:**
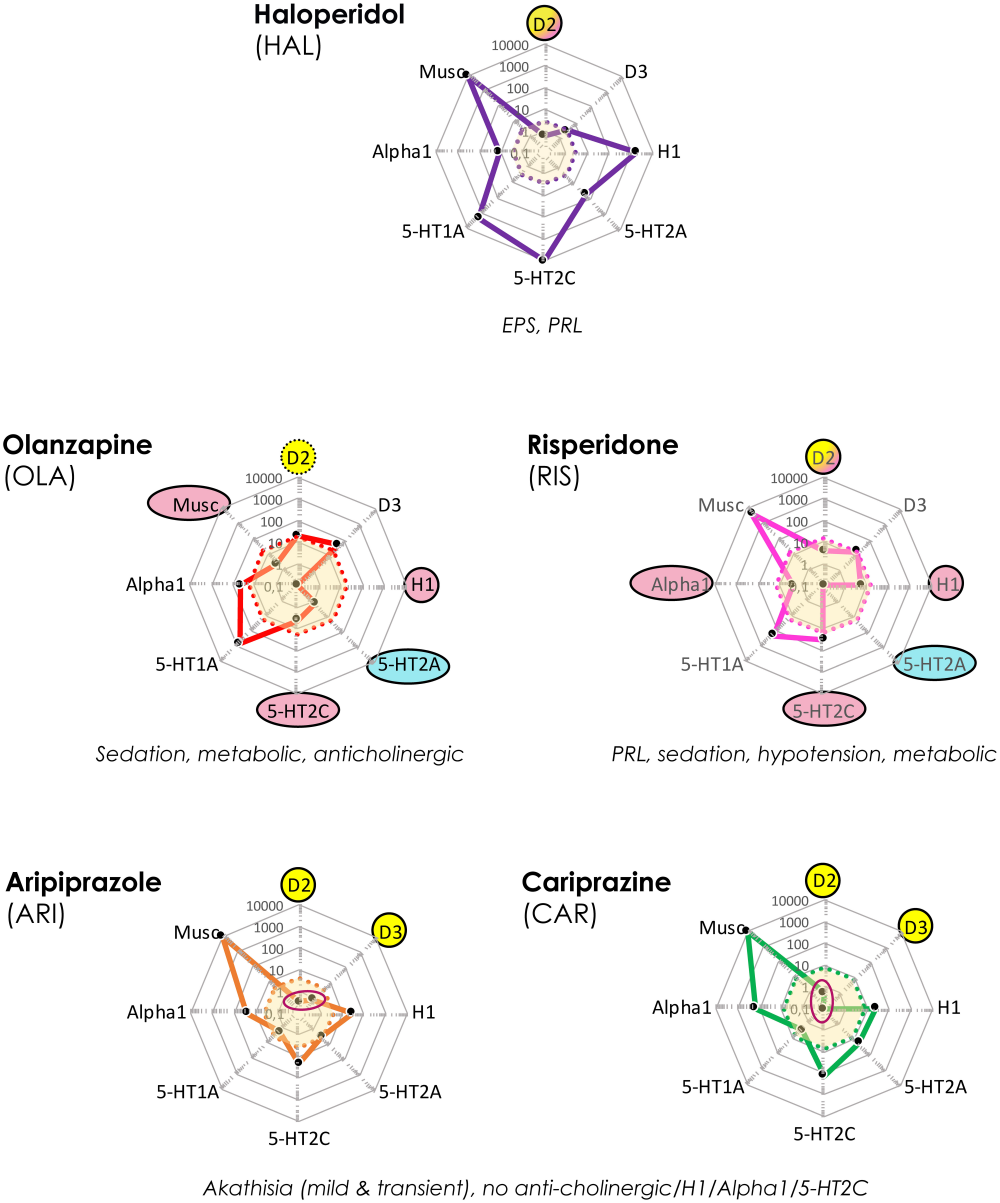
Target and associated AE profiles of HAL, OLA, RIS, ARI, and CAR. Colored lines and dots represent drug profiles based on the 8 different targets depicted at the edges of the cobweb; affinity is highest at the center, lowest at the edges (0.1–10,000 nM log scale). Dot line-enclosed shaded areas in the center represent unbound plasma levels of the compounds at efficacious therapeutic dosing. Circles and ovals depict targets likely to be affected at these levels, with colors indicating desired (yellow), accessory beneficial (light blue), and unwanted effects (red; + text below graphs). Red ovals in ARI and CAR graphs pinpoint D2 and D3 affinities. EPS, Extrapyramidal side effects; PRL, prolactin (rise). The drug cobweb profiles in this figure and Figures 5, 6 were compiled from data in public web databases and complementary literature, including drug SPC's; *viz*. human (cloned or native tissue) receptor affinities (22–24); therapeutic steady-state exposures^1^ (25); free fraction of drug plasma concentrations (26). Therapeutic steady-state exposure areas shown were obtained by converting ng/mL (25) to nM, and multiplying by the free fraction in plasma (26) for the corresponding drug.

The cobweb displays in Figure 4 reveal the markedly different pharmacological target profile patterns and accompanying clinical effect differences of HAL, OLA, RIS, ARI, and CAR. Clearly, while the “enriched” pharmacology in some antipsychotics may sometimes be an advantage [such as 5-HT2A receptors vs. motor AE; e.g., (27)], AE may also arise [such as 5-HT2C and H1 receptors vs. metabolic dysfunction; e.g., (28, 29)]. In addition, even the key D2 receptor target may bring desirable as well as unwanted clinical effects [antipsychotic action vs. EPS and hyperprolactinemia; e.g., (30, 31)]. It follows that selecting an appropriate antipsychotic medication for any individual patient should take into account not only efficacy, but the *total pharmacodynamic (PD) profile in relation to dosage and potential complementarity of neuroreceptor* action in a tentative APP approach.

### Options for Improved Clinical Outcome?

When antipsychotic drug monotherapy responding is an issue—be it for efficacy, AE/tolerability, safety, adherence, and/or other reasons—the treating physician is faced with a number of options and decisions. These may include to adjust the dose, to switch to another antipsychotic agent, and/or to consider augmentation approaches. Irrespective of which tactic is ultimately selected, apart from other clinically-based reflections there are some common basic aspects that require consideration in this situation:

*Is the reason for failure insufficient drug exposure (e.g., poor adherence, PK factors)?*

- Verify adequate antipsychotic plasma levels by therapeutic drug monitoring (TDM).

*Is the reason inadequate efficacy, intolerable AE, and/or safety issues?*

- Improve by dose adjustment, or, improve by switching to another antipsychotic.

If none of the above seem to handle the issue at hand, is APP a worthwhile approach? If so, and similarly to deliberations in a switch situation, thorough attention is recommended to (i) establish the intended therapeutic goal/s (e.g., efficacy domains, AE), (ii) select suitable antipsychotic(s) to use from the PD as well as PK perspective, taking desirable as well as unwanted outcomes into account, and (iii) work out a well-planned strategy to accomplish the goal in mind—*with patient buy-in*!

## Pharmacodynamic (PD) Considerations for APP

### The Good, the Bad, and the…Futile…? Some APP Examples

In patients for whom APP is deemed to be a fruitful treatment strategy a thorough scrutiny of available options from a pharmacological perspective is advisable. To this end, a selection of APP options is presented below, and examined from a basic PD *vs*. predicted clinical action perspective. Keep in mind though, that the relative dose/exposure of the selected antipsychotic drugs in an APP combination determines the global response, and hence that the relative clinical benefit/AE outcome in the individual patient may vary.

### Complementary Profiles, Recommendable APP From a PD and PK Point-of-View

**Figure 5:**
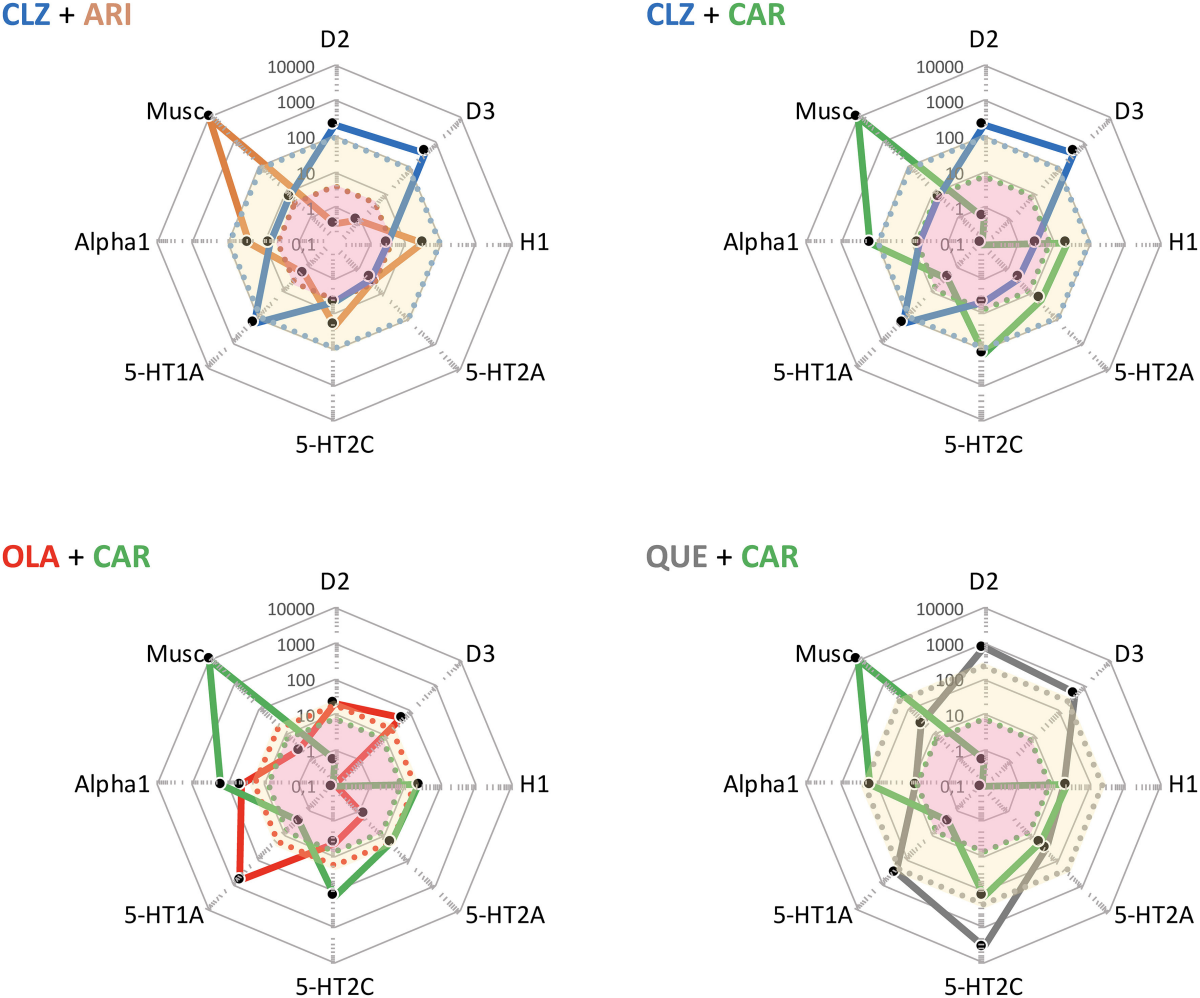
Some potentially useful APP target combinations based on FGA, SGA, and TGA. Shown are target profiles of APP combination examples based on CLZ, OLA, QUE, ARI, and CAR, displayed in a cobweb design. The colored lines joining the dots represent the target profile of the agent with corresponding color coding; e.g., blue is CLZ, and green is CAR. The dot line-enclosed shaded areas represent unbound plasma concentrations of the compounds at therapeutic dosing. For example, in the OLA + CAR case, the yellow and pink areas show unbound OLA and CAR plasma concentrations, respectively. For further explanations, see legend to Figure 4.

Refractoriness and persistent prominent negative symptomatology is a frequently mentioned basis for initiation of APP. To this end, administration of a partial DA agonist TGA together with, e.g., CLZ appears to be a particularly appealing option, as it fulfills the criteria of combining two agents with complementary pharmacodynamic (PD) as well as pharmacokinetic (PK) profiles (see, e.g., Figure 5). Indeed, ARI + CLZ is the best documented APP by far, with several studies reporting favorable outcomes both regarding efficacy (not least vs. negative symptoms), tolerability and AE [e.g., (4, 32)].

In addition, Tiihonen (33) recently reported on various APP vs. monotherapy variants with regard to risk for psychiatric rehospitalization in a nationwide Finnish historical cohort (>62,000 patients) of adult schizophrenia. They found that the CLZ + ARI combination conferred the strongest protection against rehospitalization (hazard ratio, HR = 0.42). Interestingly, among the 10 top options (lowest HR) in this regard nine were APP, seven of the APP included CLZ, and the only monotherapy was CLZ—attesting to APP usefulness, as well as to the distinctive position of CLZ in schizophrenia treatment.

Interestingly, as seen in Figure 5, the complementarity in neuroreceptor target profile patterns accomplished with a CLZ + ARI APP may be mimicked to a great extent also by CLZ + CAR, OLA + CAR, and QUE + CAR combinations. From the PD standpoint it would appear reasonable to assume that the potent D2 and D3 (and possibly also 5-HT1A) partial agonist properties of CAR will—similarly to ARI (see, above)—result in a therapeutically advantageous APP *via* complementation of a relative lack of strong interactions with these targets in CLZ, OLA, and QUE. In fact, CAR may provide a particularly interesting choice, given its prominent D3 affinity and proven clinical effect against primary negative (34) and cognitive (35) symptoms, as well as extended PK half-life (36). Although only limited clinical data with CAR in APP are hitherto available (*vide infra*), it may be hypothesized that augmentation with this agent might improve efficacy, counterbalance sedative and metabolic AE issues while maintaining the low EPS propensity of CLZ, OLA, and QUE, but also potentially elicit (typically mild, transient) akathisia. It has been suggested that 5-HT2A antagonist may be a valuable option to the β-adrenoceptor blocker propranolol against antipsychotic-induced akathisia (37). Whether or not such a component in CLZ, OLA, and QUE may serve to attenuate any akathisia triggered by CAR however remains to be established.

### Conceivable, but Theoretically Less Attractive—or Even Futile?—APP Combinations

**Figure 6:**
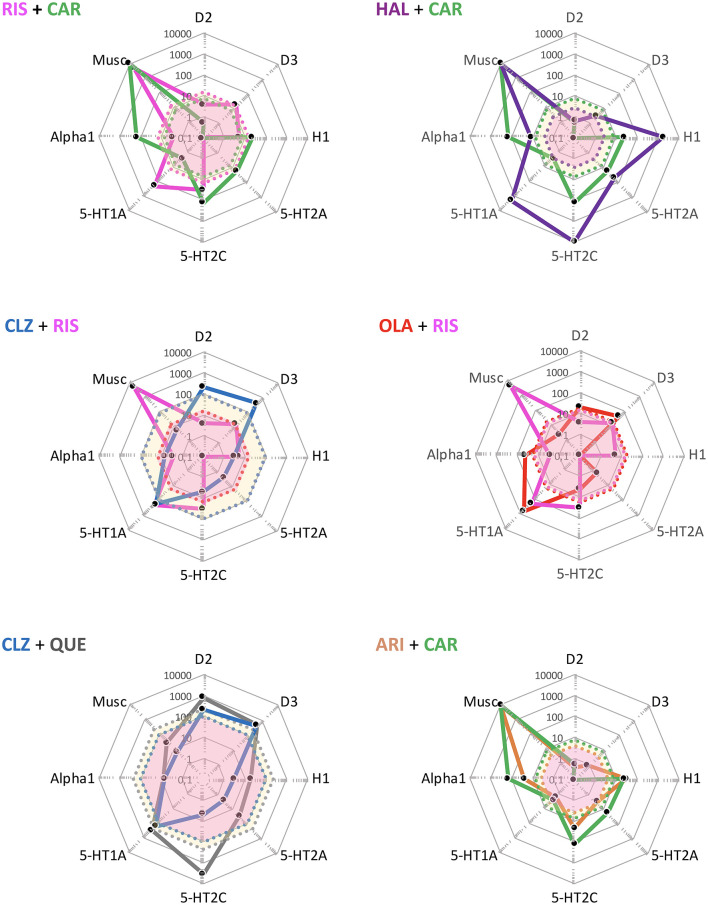
Some theoretically suboptimal, less preferable—or even futile—APP target combination examples based on FGA, SGA, and TGA. Shown are target profiles of APP combination examples based on CLZ, RIS, HAL, CAR, QUE, OLA, and ARI displayed in a cobweb design. For further explanations, see legend to Figures 4, 5.

The RIS + CAR- and HAL + CAR-based APP options are possible, though pharmacologically more complex possibilities (Figure 6). Firstly, the D3 receptor partiality of CAR adds a complementary target effect, presumably advantageous from the negative and cognitive symptomatology viewpoint [e.g., (34, 38, 39)]. However, like CAR both RIS and—in particular—HAL possess appreciable D2 receptor affinities, thereby significantly occupying such sites at therapeutic dosing. In turn, this means that the overall clinical outcome of such combinations with CAR will depend on the relative dose (/concentration) ratio between RIS or HAL and CAR, and thus be more difficult to generalize and forecast. It is possible that the high affinity and partiality of CAR at the D2 (and 5-HT1A) sites may contribute to a lower risk for RIS- and HAL-induced EPS and hyperprolactinemia [see, e.g., (27, 30, 31)]. On the other hand, it is also conceivable that because of their moderate-to-high affinity D2 receptor blockade RIS or HAL may partially counter (even obliterate?) the partial D2 receptor agonism-mediated therapeutic benefits of CAR. The differences in drug half-lives among RIS and HAL vs. CAR (*vide infra*) may also add to these complexities, thereby contributing to variability in the therapeutic outcome across the 24 h cycle [see, e.g., (14)]. Taken together, it would appear that finding the optimal dosing for these combinations may be challenging, and thus it is likely that a switch from RIS or HAL to CAR would in fact be more preferable.

APP combinations like CLZ + RIS, and OLA + RIS appear pharmacologically less desirable. Thus, whereas the poor D2 affinity of CLZ may be complemented by the higher D2 affinity of RIS, the latter agent (together with its active metabolite paliperidone; PAL) is more liable to cause EPS and hyperprolactinemia. Moreover, as both agents possess significant alpha1-adrenoceptor and H1-receptor antagonism, a CLZ + RIS APP intervention may result in an enhanced acute (orthostatic) hypotensive action as well as risk for accentuated sedation [see, e.g., (40)]. A similar reasoning may apply to the OLA + RIS combination, where any possible benefits of supplemented D2 receptor blockade may be potentially outweighed by an increased AE liability mediated by the very same site (e.g., prolactin rise, increased EPS risk), but also by other targets—as in the CLZ + RIS discussion above. It would appear that within the limitations of available reports (mostly from open, small, short-term, unblinded, non-RCT studies, or case series), clinical outcome data (efficacy and AE) with the aforementioned APP approaches do not generally demonstrate enhanced efficacy but largely concur with the pharmacology-derived reasoning above (41–47).

Among even more *questionable* (or from a pharmacological perspective, even futile) APP combinations are CLZ + QUE, and ARI + CAR. CLZ and QUE are both rather poor D2 receptor antagonists, and share many other target properties as well (e.g., antagonism of alpha1, muscarinic, H1 sites; see, Figure 6). Thus, the pharmacology-based likely lack of potential for efficacy improvement, together with a possible/probable accentuation of AE liabilities (e.g., sedation, CV, and QTc risks) renders this a pointless APP combination exercise. Only limited clinical data with the CLZ + QUE combination are found in the literature, but appear consistent with the pharmacology-based considerations given (48). The pharmacological profiles of CAR and ARI are by and large overlapping, with the exception that CAR has the decidedly higher D3 receptor affinity of the two [e.g., (22)]. It follows that an ARI + CAR-based APP combination would be futile, whereas when the D3 receptor partiality of CAR is a desired therapeutic property a switch from ARI (to CAR) might be a feasible option. To the best of my knowledge, there are no clinical reports from trials with this latter APP combination.

Taken together, for the reasons discussed above neither of the aforementioned APP combinations (illustrated in Figure 6) are ideal choices from a pharmacological perspective.

## Pharmacokinetic (PK) Considerations in APP

Antipsychotics discussed in this account are metabolized by CYP1A2, CYP3A4, CYP2D6, and/ or CYP2C19 (see, Table 3). With regard to drug-drug metabolism interactions (DDI), such issues appear relatively rare with agents commonly found on the APP scene. However, notable metabolism-derived examples include changes in plasma concentrations as a result of altered smoking habits in a patient. Smoking is an inducer of CYP1A2, and may as a result thereof lead to lower-than-expected plasma levels of CLZ and OLA, in turn calling for dose adjustment of these antipsychotics (49). Conversely, (involuntary) cessation from smoking, e.g., when a patient is hospitalized, could lead to too high exposure from agents like these, if the dosage is not correspondingly amended (*NB:* it is components in the smoke—not the nicotine—that mediates the induction of the CYP1A2 enzyme; thus, nicotine substitution approaches like patches/chewing gums may lessen abstinence issues from cigarette smoking).

**Table 3:** Examples of commonly used antipsychotics, their t_1/2_, and main metabolic enzymes.

**Drug**	**Approximate t_1/2_, h**	**CYP subtype**
Haloperidol (HAL)	21	3A4 (2D6)
Olanzapine (OLA)	33	1A2 (2D6)
Clozapine (CLZ)	12	1A2, 3A4, 2C19, (2D6)
Risperidone (RIS)	3 (~20; 9-OH)*	2D6, 3A4
Quetiapine (QUE)	6-7	3A4 (2D6)
Aripiprazole (ARI)	70	3A4, 2D6
Cariprazine (CAR)	70 (~400; DDC)^†^	3A4 (2D6)

*Data extracted from corresponding drug SPC's and the literature [see, i.a., (25)]*.

* *t_1/2_ of active metabolite to RIS; 9-OH-RIS = paliperidone (PAL)*.

† *t_1/2_ of active metabolite to CAR; DDC = di-desmethyl-CAR*.

Inhibition of drug metabolism enzymes (CYP1A2: e.g., fluvoxamine; CYP3A4: e.g., carbamazepine, ketoconazole, and grapefruit juice; CYP2D6: e.g., fluoxetine; CYP2C19: e.g., paroxetine) may result in significant DDI through an impact on the elimination—and thereby plasma concentrations—of antipsychotics metabolized *via* the corresponding pathways (see, Table 3). Dose (or drug) adjustments may therefore be necessary. Many antipsychotics are also substrates and inhibitors of the P-glycoprotein (P-gp) drug transporter [e.g., OLA, RIS, and ARI, but not CLZ and QUE; (50)]. While a P-gp-derived DDI between agents in an APP combination may thus theoretically alter plasma and brain concentrations of other substrates, actual patient outcomes are less clear (50); this conceivable DDI risk should nonetheless be kept in mind (For further details on putative PK-derived DDI, please consult relevant drug SPC's).

When choosing antipsychotics to combine in an APP regimen, it is prudent from the PK view to combine antipsychotics that differ in half-life (i.e., a short-acting plus a long-acting agent), time to peak concentration, and ideally also elimination pathway. Hence a better control of fluctuations in DA receptor occupancy may be attained, and some “buffer” capacity provided to promote compliance and prevent relapse in a situation with outpatients that may show erratic medication adherence.

The use of long-acting formulation injection antipsychotics (LAI) is common in schizophrenia treatment, and while LAI-based APP appeared more prevalent before the 1990's than in the 2000's (2), it is still in frequent use (51). The main reason for LAI use overall appears to be maintained compliance, particularly in difficult-to-treat patients (2). Needless to say, whereas the PK properties are distinctly different in oral and LAI formulations of the same drug, the PD target profiles remain identical. LAI is intended to produce a more flat, stable, PK profile vs. the drug targets involved. In APP approaches, the stable target occupancy advantage may be lessened when LAI treatment is accompanied by concomitant oral dosing, either by another antipsychotic, or by the same agent as given by LAI tentatively, i.a., to obtain a more fine-tuned dosing regimen; (1), which may in turn also have implications toward the strength of PD effects across time [discussed, e.g., by (14)]. This said, the variability in target occupancy introduced by LAI-based combinations with oral antipsychotics would appear less significant using agents with complementary target profiles (see, Discussion above), like, e.g., combining QUE or CLZ with TGA like ARI or CAR. Taken together, the very same pharmacological principles would thus appear to be valid regardless of whether oral + oral or LAI + oral APP treatments are considered.

Notably in this context, CAR gives rise to a very long-acting active metabolite (DDCAR, Table 3; (36) with essentially matching target affinities and profile to its parent compound (52). At steady-state, CAR may thus be viewed as a “long-acting oral” treatment for schizophrenia, valuable also from a compliance and relapse perspective (53).

## Theoretical Usefulness of the APP Examples Discussed

Table 4 summarizes the APP examples illustrated and discussed above in Figures 5, 6, with brief pharmacology-based overview comments and recommendations.

**Table 4:** Summary of theoretical pharmacological usefulness of APP examples discussed.

**Overall rating**	**APP combination**	**Comments**
Fine	CLZ + CAR	Complementary PD and PK profiles—potential for improved efficacy as well as AE outcome + short- and long-acting agent combination (contributing to compliance)
	CLZ + ARI	
	OLA + CAR	
	QUE + CAR	
Conceivable, but possible issues	RIS + CAR HAL + CAR	Some potential for improvement, but complex PD interaction—challenging to optimize doses for efficacy and AE benefits
	CLZ + RIS OLA + RIS	Doubtful efficacy improvement; increased AE burden
Futile	CLZ + QUE	PK as well as PD profile overlap
	ARI + CAR	

### APP: Reduction of AE?

Antipsychotics clearly differ in AE liabilities and severity, with TGA being generally more benign than FGA and SGA [e.g., (54)]. This also applies regarding the propensity to induce weight gain and accompanying metabolic AE, with the SGA OLA and CLZ displaying the most, and TGA agents like ARI and CAR the least, harmful profiles (55). Additionally, marked antipsychotic drug heterogeneity in prolactin-raising and sedation-inducing properties occur (40, 56). From the APP perspective it is notable that add-on treatment with TGA can significantly attenuate OLA- and CLZ-induced AE like the aforementioned (57–60).

### Case Reports—CAR Add-On to CLZ or QUE

**Figure 7:**
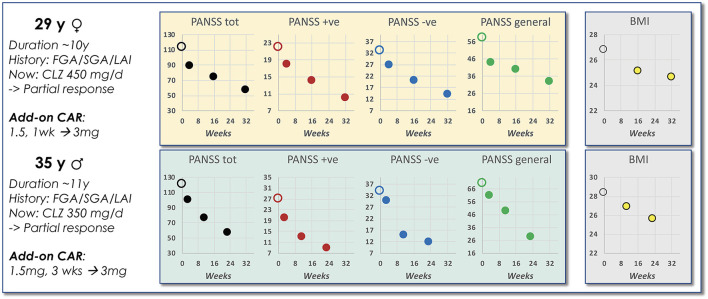
Data from a case report of CAR add-on to CLZ treatment. Shown is a brief schizophrenia treatment history of a female and a male patient prior to APP add-on with CAR to ongoing CLZ treatment. The accompanying graphs illustrate PANSS scoring and BMI observations evolving over time following start of the CAR add-on intervention [data extracted from De Berardis et al. (16)].

So far, only very limited data on APP involving CAR is available. However, De Berardis et al. (16) recently reported on CAR add-on in two patients, with comparable illness and medication backgrounds, but only partially responding to CLZ treatment. In both of these cases the CAR addition within 6–8 months brought about a marked remission across symptom domains as scored by PANSS (Figure 7). Interestingly—and in line with the above predictions—body mass index (BMI) dropped an impressive ~3 units from baseline over the same period for both patients. These findings thus support the view that the APP combination of an antagonist and a partial DA agonist antipsychotic agent with complementary PD profiles and short- vs. long-acting PK properties may result in an advantageous outcome both with regard to the desired efficacy and unwanted AE features of the treatment.

Interestingly, a recent single-patient case report suggests that CAR plus QUE may also be an attractive APP option (18). The combined treatment with these two antipsychotics resulted in successful alleviation of cognitive and negative impairments in a young male patient, whom previously had been through a variety of but partially efficacious FGA and SGA regimens. Intriguingly, and in line with other recent case studies, the CAR + QUE APP was associated with an abrupt cessation of smoking, and curbed use of recreational drugs, tentatively indicative of anti-craving effects. In support of this speculation, combined CAR + QUE treatment was reported to markedly attenuate alcohol craving and bring about lasting symptom stability in a bipolar I patient (61). Further, CAR monotherapy has also been reported to result in abrupt remission from persistent methamphetamine psychosis and improved positive as well as negative symptomatology in a treatment-naïve male patient (62), and to benefit two cases of schizophrenic patients with other substance use disorders (63). These clinical observations are consistent with preclinical literature and theory suggesting that D3 receptors may be implicated in drug dependence issues (64–66).

### Partial Agonist Effects on Symptoms and (Antagonist-Derived) AE in APP Approaches

What would be the potential PD mechanistic substrate/s underlying partial agonist-induced improvements of core symptoms and AE when incorporated into an APP approach? From a theoretical viewpoint it appears plausible that the effects on positive and negative symptomatology involve partial agonism at the DA D2 and D3 receptor sites (Table 5). A direct D2 receptor interaction is also highly likely to explain the normalization of antagonist-induced hyperprolactinemia. However, the reported beneficial effects on anthropometric and metabolic issues, as well as the offsetting of sedation is hypothesized to be the result of counterbalancing neuronal circuits rather than direct competition between drugs at a particular target (Table 5). That is, the blockade of, i.a., H1 and 5-HT2C receptors in some brain regions/neurocircuitries drives the weight, metabolic parameters, and sleepiness in one direction, whereas partial D2 agonism drives them the opposite way. Indeed, generally, agents in the TGA partial DA agonist class are considered less metabolically adverse [see, e.g., (55)], and also more “activating” and less “sedating” as compared to FGA and SGA (67).

**Table 5:** Summary of putative clinical impact of APP treatment involving partial agonists.

**Issue**	**Type**	**Putative clinical impact (target/s)**
Positive symptoms	Desired	Improvement (partial D2)
Negative symptoms	Desired	Improvement (partial D3)
Hyperprolactinaemia *(RIS, HAL, PAL)*	AE	Attenuation (partial D2)
Weight/metabolic *(e.g., OLA, CLZ)*	AE	Improvement *(interaction partial D2 vs. H1/5-HT2C?)*
Sedation *(e.g., OLA, RIS)*	AE	Improvement *(interaction partial D2 vs. H1?)*

## APP: Some Brief Practical Points

Clearly, many aspects deserve attention when considering initiation of APP. Pae (9) discussed some practical points and tips in a recent review; some of these points are extracted and briefly summarized below:

- Make it clear (to self and patient) why you wish to use APP
- Use measurement-based APP (PANSS, CGI, other scale/s) to monitor effects over time, and increase the ability to link desired/adverse effects to the drug/s in question
- Consider APP for patients with ≥2 failed monotherapy trials, including a trial with CLZ
- Apply rational pharmacological APP reasoning; paying attention to both PD target and mechanism of action complementarity
- Closely monitor *total* AP dose levels, and aim to keep total dosage down
- CLZ is the best documented agent in these contexts, and should thus be one of the first antipsychotic drug options toward APP
- Consider long- plus short-acting agents in the planned APP regimen, hence also applying PK complementarity in the drug treatment

### APP: Overall Theoretical Considerations

In patients for whom APP is deemed to be a worthwhile treatment strategy, it is evident from the above that a thorough scrutiny of available options is advisable. By and large, whereas combinations of (FGA and SGA) D2 receptor antagonists may be challenging, available data discussed in this account indicate that from the pharmacological perspective selected APP, in particular based on SGA + TGA, may indeed be efficacious, tolerable, safe, as well as useful in a preventive, relapse/re-hospitalization context.

Within this APP framework, a PD comparison between the TGA:s CAR and ARI suggests that while both display high affinity partial agonist activity at the D2 receptors, CAR displays even higher affinity for the D3 than the D2 sites and is nearly 10-fold more potent than ARI at D3 receptors [e.g., (22)]. It may be hypothesized that, although similar in their efficacy against positive symptoms the appreciably stronger D3 action of CAR vs. ARI may translate to an improved profile toward primary negative symptoms—and, speculatively, also when dependence issues may be involved (see, above). Further, while both agents may elicit mild and typically transient akathisia, neither appears burdened by marked EPS, metabolic issues, prolactin rises, or sedation (68). As both agents have long t_1/2_ (CAR > ARI; see, Table 3), an SGA + TGA combination in an APP strategy setting thus also fulfills the PK aim to match a short-acting with a long-acting agent.

From an efficacy point-of-view it appears probable that APP will be prescribed for (i) patients with predominant and persistent negative symptoms, and (ii) patients with residual positive symptoms; e.g., patients with chronic auditory verbal hallucinations, only partly alleviated by antipsychotic monotherapies. Unfortunately, available literature does not seem to shed very much light on whether a particular type of APP would be preferred for one vs. the other of these forms of enduring issues. However, as a recommended Guideline sequel to ≥ 2 failed monotherapy trials, it appears logical that CLZ would be highly prevalent in any strategies to deal with persistent residual symptoms—irrespective of domain. While agents with high affinity and antagonism or partial agonism at the D2 receptors are commonly used to attenuate positive symptoms the negative/cognitive domains appear overall less susceptible and more difficult to reach, even with CLZ. From a pharmacological standpoint partial agonist TGAs like ARI or CAR should be useful to boost efficacy of CLZ, although so far high-quality data are supportive only for ARI with respect to negative symptoms [discussed above, see (4)]. The notable efficacy of CAR monotherapy in this latter indication (34, 35) may possibly suggest a further edge of this agent in APP when negative/cognitive issues dominate the clinical picture. Until further studies to assess this prediction, it however must remain purely speculative.

## Strengths and Limitations

While the above pharmacology-derived assessments are based on an extensive appraisal of antipsychotic literature data, it should be pointed out that the profile comparisons and associated APP recommendations are based on free drug concentrations in *plasma*—used as a proxy estimate for CNS (and, in part, peripheral) target interactions. This said, there does not seem to be any clinical data that directly contradict the interpretations put forward. On the contrary, APP studies reported in the literature do in fact seem quite aligned with the pharmacodynamic target-based analysis offered.

## Summary and Conclusions

In conclusion, APP treatment may be useful in selected patients when switch is not desired or feasible, but is NOT to be applied for ROUTINE use. High-quality studies, with proper pharmacological resolution, are needed toward the generation of evidence-based strategy guidelines for APP treatment of schizophrenia when required in clinical practice [see, (69)]. If an APP combination intervention is considered and initiated, it should

- only be used after ≥2 failed monotherapy trials (adequate dose and duration)
- be based on agents with *complementary* neuroreceptor profiles
- take PK, safety (regular health checks) and tolerability into proper consideration
- always allow sufficient time to establish post-combination treatment outcome

In closing: any APP regimen should be based on drugs that are complementary, beneficial from an efficacy/AE outcome perspective, and follow a clear therapeutic rationale, avoiding PK as well as PD risks. The chosen antipsychotic combination should also focus on the prioritized symptom domains, while avoiding dispensing unnecessary, ineffective or redundant psychotropic agent exposure to individuals with schizophrenia. Against this backdrop it would appear that APP based on add-on with Third Generation Antipsychotics, TGA (e.g., CAR or ARI) may be particularly useful, together, e.g., with CLZ. It should be kept in mind though, that although APP may be both feasible and beneficial, monotherapy is still the preferred state. Consequently, if possible, switching options should always be thoroughly considered before embarking on a combination treatment intervention.

## Data Availability Statement

The original contributions presented in the study are included in the article/supplementary material, further inquiries can be directed to the corresponding author/s.

## Author Contributions

SH was solely responsible for the conceptualization, subject research and interpretations, writing, editing, and approval of the submitted version of the article.

## Funding

The writing of this report was in part sponsored by Recordati, but the company had no influence on data collection, analysis, content, or interpretations. Recordati also provided funds for the open access publication fees.

## Conflict of Interest

Over the last 3 years, SH has received honoraria from Lundbeck, Otsuka, Gedeon Richter, and Recordati for scientific talks and/or participation in advisory boards. He does not hold any shares or has financial interest in any of the companies marketing the antipsychotic agents discussed in the paper. SH is a self-employed independent consultant with his own company Pharmacilitator AB (Inc.).

## Publisher's Note

All claims expressed in this article are solely those of the authors and do not necessarily represent those of their affiliated organizations, or those of the publisher, the editors and the reviewers. Any product that may be evaluated in this article, or claim that may be made by its manufacturer, is not guaranteed or endorsed by the publisher.

## Footnotes

^1^*Disclaimer*: Needless to say, the size of the exposure area will vary depending on the actual dose, but also individual variation. The area limit shown is created from population-based therapeutic steady-state exposures, defined as “*upper limit above which tolerability decreases or above which it is relatively unlikely that therapeutic improvement may be still enhanced (25)*”.
